# Supplementary material for: Magnolol Supplementation Alters Serum Parameters, Immune Homeostasis, Amino Acid Profiles, and Gene Expression of Amino Acid Transporters in Growing Pigs
Source: Int J Mol Sci. 2023 Sep 11;24(18):13952. doi: 10.3390/ijms241813952 (PMC10530316; doi:10.3390/ijms241813952)
Supplement: Supplementary file 1 [file ijms-24-13952-s001.zip › ijms-2567989-supplementary.pdf]

Table S1. Feed ingredient and nutrient levels of the basal diet (%, as−fed basis).

Item	content
Ingredients, %	
Corn	63.5
De−hulled soybean meal (46%)	26
Wheat bran	5
Soybean oil	2
L−Lys HCl (98%)	0.23
DL−Methionine (99%)	0.03
L−Threonine (98.5%)	0.04
$CaHPO_4$	1
Limestone	0.9
NaCl	0.3
Vitamin and mineral premix[1]	1
Total	100.00
Calculated nutrient composition[2], %	
DE[3], MJ/kg	14.21
Ca	0.66
Available P	0.31
Standardized ideal digestible AA, %	
Lys	1.14
Met	0.33
Thr	0.73
Trp	0.22
Analyzed nutrient composition[4]	
Dry matter, %	86.39
Crude protein, %	17.69
Crude fat, %	2.28
Crude ash, %	8.28

[1]Supplied per kilogram of complete diet: vitamin A, 15,500 IU; vitamin D3, 3,500 IU; vitamin E, 37.5 mg; vitamin K_3, 6.25 mg; vitamin B_1, 3.75 mg; vitamin B_2, 12.5 mg; vitamin B_6, 10 mg; vitamin B_{12}, 0.05 mg; niacin, 50 mg; calcium pantothenate, 18.75 mg; folic acid, 1.25 mg; biotin, 0.1 mg; Cu ($CuSO_4·5H_2O$), 9.6 mg; Fe ($FeSO_4·H_2O$), 78 mg; Zn ($ZnSO_4·H_2O$), 54 mg; Mn ($MnSO_4·H_2O$), 42 mg; I (KI), 0.42 mg; Se (Na_2SeO_3), 0.29 mg; Co ($CoCl_2$), 0.24 mg. [2]Values were calculated using the feed composition and nutritive values provided by Feed Database in China (2020). [3]DE, digestible energy. [4]Analyzed results obtained according to the analytical methods in AOAC (2007).

Table S2. Primer sequences used for qRT−PCR assay

Genes	GenBank ID	Primer sequence (5′→3′)	Product size
**β−actin**	XM_003357928.1	Forward: GGACTTCGAGCAGGAGATGG	233
		Reverse: GCACCGTGTTGGCGTAGAGG	
SLC1A3	XM_021076550.1	Forward: GCAAGCACTCGTCACAGCTC	115
(*EAAT1*)		Reverse: GGAGACGAATCTGGTGACA	
SLC7A8	XM_003128550.5	Forward: ACTACCTCTTCTATGGCATCAC	111
(*LAT2*)		Reverse: GCAAGTAGATGATGGGGAACAG	
SLC38A2	XM_003126626.5	Forward: TTCATTCTTCCATCTGCCTTC	154
(*SNAT2*)		Reverse: GGGCATTGTGTACCCAATC	
SLC1A5	XM_003127238.4	Forward: CGATTCGTTCCTGGATCTTG	81
(*ASCT2*)		Reverse: TAGGACGTCGCGTATGAG	
SLC7A1	AY371320	Forward: GTCGGTTGCAAAGACCATTT	329
(*CAT1*)		Reverse: GAGCGGTGCTGACAACAGTA	
ASS1	XM_005660523	Forward: CCCTCACTTTGCCCATCTCT	163
		Reverse: CCCTACCCTTCCGTTTGCT	
SLC3A1	NM_001123042.1	Forward: CAATGCAGTGGGACAACAG	158
(*rBAT*)		Reverse: GGCGTGAAGCAAACTTAATTC	
SLC7A7	NM_001110421.1	Forward: CTCTGCTGTTCAATGGTCTC	125
($y^{+}LAT1$)		Reverse: ATAGAGCTGACCCACGATAG	
SLC7A9	EF127857.1	Forward: CGGAGAGAGGATGAGAAGT	562
($b^{0,+}AT$)		Reverse: GCCCGCTGATGATGATGA	

Note: β−actin was used as an internal control. *SLC38A2* (*SNAT2*), solute carrier family 38 member 2; *SLC1A5* (*ASCT2*), solute carrier family 1 member 5; *SLC7A9* ($b^{0,+}AT$), solute carrier family 7 member 9; *SLC3A1* (*rBAT*), solute carrier family 3 member 1; *SLC1A3* (*EAAT1*), solute carrier family 1 member 3; *SLC7A8* (*LAT2*), solute carrier family 7 member 8; *SLC7A1* (*CAT1*), solute carrier family 7 member 1; *SLC7A7* ($y^{+}LAT1$), solute carrier family 7 member and *ASS1*, ar−gininosuccinate synthetase 1.

Table S3. The serum differential metabolites were identified according to the standard of VIP > 1.0 and *P* < 0.05 between the Con and Mag groups.

Metabolites	VIP [1]	*p*–value	KEGG [2]	Trend (Mag [3] vs. Con [4])
Estrone−3−glucuronide	2.65	0.000	C11133	Up
Adipic acid	2.02	0.001	C06104	Down
Jasmonic acid	2.07	0.002	C08491	Down
Alpha−hydroxyhippuric acid	2.16	0.003	—	Down
3−hydroxybenzoic acid	1.87	0.010	C00587	Down
2−phenylacetamide	1.77	0.011	C02505	Up
Thymine	1.81	0.012	C00178	Down
L−arginine	1.73	0.014	C00062	Up
Phenylacetylglycine	1.71	0.018	C05598	Down
Acetophenone	1.57	0.020	C07113	Down
N6,N6,N6−trimethyl−*L*−lysine	1.68	0.026	C03793	Down
Creatinine	1.59	0.029	C00791	Down
Ribonolactone	1.74	0.030	C02674	Down
2−oxoglutaric acid	1.63	0.034	C00026	Down
Hippuric acid	1.71	0.035	C01586	Down
N−a−acetyl−*L*−arginine	1.44	0.036	—	Up
Creatine	1.60	0.036	C00300	Down
2−hydroxyquinoline	1.64	0.043	—	Down
Alpha−hydroxyisobutyric acid	1.50	0.044	—	Down
4−nitrophenol	1.48	0.046	C00870	Down

[1]VIP: Variable importance in the projection. [2]KEGG: Kyoto Encyclopedia of Genes and Genomes. [3]Mag: a basic diet food with 0.04% magnolol. [4]Con: a basic diet.
